# 139. Development of cefiderocol resistance in an *Enterobacter hormaechei* strain following prolonged antibiotic exposure

**DOI:** 10.1093/ofid/ofac492.217

**Published:** 2022-12-15

**Authors:** Rachel Medernach, William J Moore, Sophia Nozick, Maha M Alamri, Kendall Kling, Michael G Ison, Qi Chao, Kelly E R Bachta, Nathaniel J Rhodes, Egon A Ozer, Alan R Hauser

**Affiliations:** Northwestern University Feinberg School of Medicine, Chicago, Illinois; Northwestern Medicine, Chicago, Illinois; Northwestern University, Chicago, Illinois; Northwestern Memorial Hospital, Chicago, Illinois; Northwestern University Feinberg School of Medicine, Chicago, Illinois; Northwestern University Feinberg School of Medicine, Chicago, Illinois; Northwestern Memorial Hospital, Northwestern University Feinberg School of Medicine, Chicago, Illinois; Northwestern University, Chicago, Illinois; Midwestern University, Downers Grove, Illinois; Northwestern University Feinberg School of Medicine, Chicago, Illinois; Northwestern University, Chicago, Illinois

## Abstract

**Background:**

Cefiderocol (FDC) is a novel antimicrobial agent used for multi-drug resistant Gram-negative pathogens. To date, reports of mutations in β-lactamase and siderophore complex genes have been described and may contribute to FDC resistance. This case describes a New Dehli metallo-β-lactamase (NDM)-producing strain of *Enterobacter hormaechei* that developed FDC resistance following antibiotic exposure.

**Methods:**

Serial respiratory and blood cultures were collected from a lung transplant recipient throughout 72 days of hospitalization. Confirmatory susceptibility and combination minimal inhibitory concentration (MIC) testing were performed using broth dilution and E-test assays. Short-read sequencing libraries were prepared using a seqWell plexWell 96 kit, and whole-genome sequencing was performed using the Illumina NovaSeq platform. Reads from the sample genomes were aligned to the chromosome and three plasmid sequences of reference genome ENCL48880.

**Results:**

Four serial respiratory *E. hormaechei* isolates and one blood isolate were evaluated. Although initial isolates were susceptible to FDC (MICs 1-2 µg/mL), two respiratory isolates cultured after 41 days of FDC therapy had MICs of 128 µg/mL. The blood isolate remained FDC susceptible despite respiratory resistance. The combination of ceftazidime/avibactam and aztreonam was determined to be active via synergy MIC testing in all isolates, and aztreonam therapeutic drug monitoring confirmed an adequate dosing strategy. Whole-genome sequencing revealed no nonsynonymous single nucleotide variants (SNVs) within the chromosomes but identified a deletion of a large urease island in the resistant isolates. In four of the five isolates, a plasmid (p48880_mcr) was identified and analyzed for possible contributions to FDC resistance.

Enterobacter Isolate Assemblies

**Figure:**
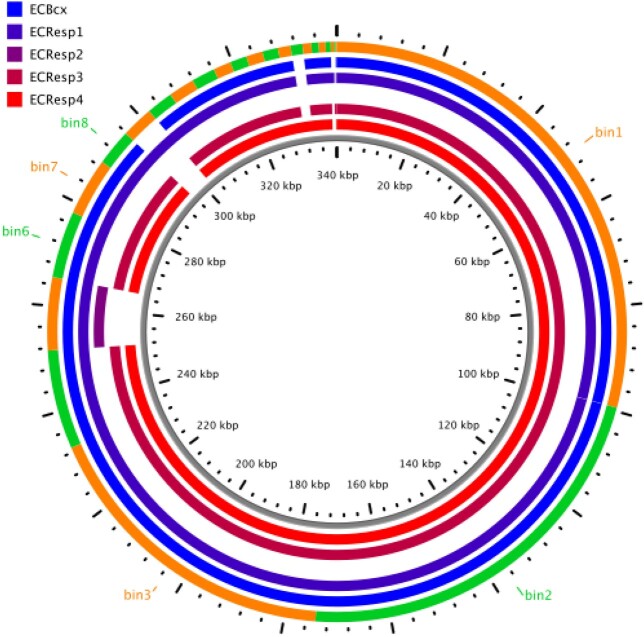

This figure demonstrates genomic assemblies from the five Enterobacter clinical isolates, noting an absence of sequence from ECResp2.

**Conclusion:**

This case demonstrates development of FDC resistance in *E. hormaechei* isolates during a 41 day course of FDC therapy. Possible causes of resistance include a large chromosomal deletion and plasmid alleles, demonstrating a potential novel mechanism for FDC resistance. Partnering molecular testing and enhanced antimicrobial stewardship should be encouraged to optimize selection of regimens and durations to prevent resistance to FDC.

**Disclosures:**

**Michael G. Ison, MD MS**, GlaxoSmithKlein: Grant/Research Support|Pulmocide: Grant/Research Support|Viracor Eurfins: Advisor/Consultant **Nathaniel J. Rhodes, PharmD, MSc**, American Academy of Colleges of Pharmacy: Grant/Research Support|Paratek: Grant/Research Support|Third Pole Therapeutics: Advisor/Consultant.

